# NEWS 2

**DOI:** 10.7189/jogh-07-010202

**Published:** 2017-06

## Agencies

### The Bill and Melinda Gates Foundation

➢ In a *Project Syndicate* article, Bjørn Lomborg praised the Bill and Melinda Gates Foundation’s strategy of focusing on three specific investment areas (vaccination, contraception and nutrition), all of which generate high returns. Eg, expanding vaccination programmes by US$ 1 billion/y to prevent childhood pneumonia and diarrhea would save 1 million lives each year, with each US$ 1 invested generating US$ 60 of human welfare. Likewise, achieving near–universal access to contraception would prevent 150 000 maternal deaths and 600 000 children from being orphaned, with a resultant boost to economic growth from the demographic dividend – generating US$ 120 for every US$ 1 invested. And improving children’s nutrition in the first 1000 days of life can return benefits of US$ 45 per US$ 1 spent, rising to US$ 166 in some countries. He contrasts this approach with the UN’s Sustainable Development Goals (SDGs), which cover these areas, but they are buried within scores of other detailed and off–topic topics such as sustainable tourism and inner–city garden access for disabled people – ie, worthy issues, but not necessarily the most vital ones and do not provide signposts for the most vital investments. He recognizes that the BMGF can focus on what works best and, unlike governments, does not face competing priorities and taxpayers’ sentiments. However, focusing on what works best saves lives – the previous Millenmium Development Goals encapsulated 8 simple, clear goals, and have saved at least 21 million lives. (Project Syndicate, 14 February 2017)

➢ The Cambridge MA firm Affinivax, with key support from the Bill and Melinda Gates Foundation, has been working on the first clinical trial of a successor to Pfizer’s pneumococcus vaccine Prevar 13, which had US$ 6 billion turnover in 2016. Affinivax has abandoned the traditional conjugation pathway for the vaccine development, but instead uses a Multiple Antigen Presentation System, that allows the new vaccine to cover a full spectrum of 90 strains of pneumococcus instead of the 13 strains covered by Prevnar 13. Moreover, the vaccine’s efficacy is boosted by using its proteins to elicit B and T cells responses, instead of being used as a carrier. Although Affinivax has not formally declared a timeline for the clinical trials, it has completed preclinical trials and is finalising its Good Manufacturing Practice plans. (Endpoint News, 28 February 2017)

➢ A Bill and Melinda Gates Foundation–funded project to set up a Faecal Sludge Treatment Plan (FSTP) in India’s Trichy district has been challenged by the Woraiyur–Pandamangalam and Thamalavarabuyam Villages Agriculturists’ Association. FSTPs remove, treat and dispose of faecal sludge, and the purified water from the cleaning process can be used for irrigation and the sludge for fertiliser. The plant, which has received US$ 5.5 million of funding from the Foundation, has been challenged on the grounds that it violates the city’s master plan, planning consent was granted in secret, and tendering protocols were not followed. The Association filed a Public Interest Litigation petition, and the court directed the commission of Trichy to file a counter–argument. (Times of India, 7 March 2017)

➢ The Bill and Melinda Gates Foundation has committed US$ 80 million to close gender data gaps, and in *The Wire India*, Katherine Hay of the BMGF’s India office argues that data are essential for measuring progress on gender equality goals. Data allows us to know where the problems are, to better understand them and to solve them. She highlights the high rate of infant mortality in Bihar, where 4.2% of babies will die by their first birthday. This figure breaks down into 3.6% of boys and 5% of girls dying by their first birthday – a huge gap with no biological foundation. The data also uncovers other gaps, such as birth and death registration – better registration would have thrown up this gap much earlier. Data can also help reveal why fewer girls are surviving, eg, different immunisation rates, poorer nutrition, or lower care seeking and levels of spending. Understanding the gap can present solutions, arising from evaluations what has worked elsewhere. This allows funds to be directed toward effective programmes, although data are lacking on what works well at scale. Evidence from data will help policy–makers in India, and elsewhere, to drive change and tell them how to do better. (The Wire India, 8 March 2017)

➢ In an interview with BBC News, Bill Gates commended pharmaceutical companies for their role in donating drugs to combat neglected tropical diseases. Globally, 1 billion people were treated for at least one tropical disease in 2015, and companies have donated 7 billion treatments since 2012. Every year, 170 000 people die from neglected tropical diseases, but their biggest impact lies in the resultant disabilities. Speaking from a meeting in Geneva, where new commitments of US$ 812 million from governments, drug companies and charities were made to continue the fight against neglected tropical diseases, Mr Gates highlighted some of the campaign’s successes. These include substantial falls in lympathic filariasis and sleeping sickness, and the near–elimination of guinea worm. He expressed confidence that pragmatism will prevail on maintaining the USA’s strong development aid budget, and has already had talked with President Trump on the US’s critical role in progress on HIV, malaria and reproductive health, and how strong health systems can stop pandemics. He lauded the UK government’s recent announcement that it would double support for fighting neglected tropical diseases, saying “the UK is a critical donor. As somebody who’s very measurement–oriented, I find that partnering with the UK on these health–related areas is a great way to spend money and lift these countries up.” (BBC, 19 April 2017)

### The GAVI Alliance

➢ GAVI, along with Google.org, and the Bill and Melinda Gates Foundation, are providing funding to Nexleaf Analytics, a Los Angeles–based start–up that produces wireless sensors and data analytics tools such as ColdTrace – a remote temperature monitoring technology that protects vaccines. The funding is part of GAVI’s INFUSE scheme, to accelerate support in Innovation for Uptake, Scale and Equity in Immunisation. With this funding, Nexleaf will develop an analytics framework, gathering data from the countries its technology reaches, to share data with governments seeking to make evidence–based decision–making. The main attraction with Nexleaf’s approach is how it prioritises data to drive decision–making and ensuring that data are actionable and useful, rather than the technology itself. This is part of GAVI’s strategy of innovating to strengthen health systems, in this case modernising cold chains. “A lot of new technologies for gathering data are either on the market or in development, from smart fridges to temperature monitors. Our experience working with everyone from nurses in clinics to national ministries of health, and with making the real time data available in formats that servce them, is what Nexleaf brings to the table,” said Martin Lukac, co–founder and chief technology officer of Nexleaf. (Devex, 18 January 2017)

➢ GAVI and Deutsche Post DHL announced a global partnership to help improve vaccine supply chains in GAVI–supported countries, to overcome the constraints placed by outdated supply chains. Deutsche Post DHL’s logistical expertise in health care and life–sciences, combined with its global transportation network, will help to make vaccine supply chains more efficient, helping to increase coverage, reduce vaccine wastage and protect availability and potency – ultimately saving lives. It is essential to improve supply chains, as vaccine volumes grow and weak health systems struggle to cope with expanded immunisation programmes. Kenya will be the first country to trial the new partnership, with its Ministry of Health testing a dedicated transportation management solution to distribution vaccines throughout the country. The partnership dovetails with GAVI’s strategy of forming clusters of private–sector alliances to address coverage and equity bottlenecks, accelerating progress and achieving lasting impact. “Robust vaccine supply chains are a vital part of building strong health systems, so that children, parents and communities can be reached wherever they live with life–saving vaccines. DHL’s expertise in healthcare delivery and its footprint in sub–Saharan Africa will help the partnership develop and test new innovative solutions aimed at increasing the health impact in GAVI–supported countries,” said Seth Berkley, CEO of GAVI. (Global Trade Magazine, 29 January 2017)

➢ Results for Development, a not–for–profit organization based in Washington DC, USA, released its new resource guide to provide practical advice for low– and middle–income countries (LMICs) who are planning on mobilising resources for their immunisation programmes. The guide, *Immunization Financing: A Resource Guide for Advocates, Policymakers and Program Managers,* offers 26 briefs, including 8 case studies, to assist countries in providing sustainable financing of immunisation. The guide is an update to the current *Immunization Financing Toolkit,* published by the World Bank and GAVI in 2010. Its publication is timely as many countries have pledged to increase domestic financing for vaccines and immunisation delivery, new vaccines are being developed, and many countries experiencing economic growth are moving from GAVI support toward full domestic financing of their immunisation programmes. (allafrica.com, 21 February 2017)

➢ GAVI is supporting the introduction of new coolers and refrigerators that run on solar power to keep vaccines cold. Dr Orin Levin, Director of Vaccine Delivery at the Bill and Melinda Gates Foundation, and the BMGF’s focal point of engagement with GAVI, highlighted Ghana’s success in being the first country in Africa to introduce pneumococcal and rotavirus vaccines, thereby jointly tackling two of the biggest killers of the world’s children – pneumonia and diarrhea. He notes that Ghana’s leadership in this area will pave the way for other African and Asian countries to follow suit. He commended Ghana for its strong partnership between communities and local health workers, adding that “vaccination programmes work best when health workers, traditional leaders and parents are all actively involved and understand the value of vaccines and the importance of bringing kids in on time.” He was speaking at the celebration of this year’s Vaccination Week, which coincided with the first anniversary of the Ministerial Conference on Immunisation in Africa and the ground–breaking Addis Declaration on Immunisation (ADI). (Ghana News Agency, 12 May 2017)

➢ In an op–ed piece in the *New York Times*, GAVI’s CEO, Seth Berkley, wrote of the potential global health threat posed by yellow fever, as Brazil faces an unusually large outbreak, with 715 confirmed cases, 820 suspected cases and 240 confirmed deaths. To date, these cases have been in remote, sparsely populated areas, but if it spreads to urban areas, it would be very difficult to contain. Yellow fever is transmitted by certain mosquito species, and kills more than 30 000 people each year. There is a real risk that it could spread to previously unaffected areas, such as Asia, where the presence of mosquitos and 1.8 billion unvaccinated people in a densely–populated region is a potentially catastrophic combination. Cities in Brazil are intensively vaccinating their citizens, in the hope of reaching 12 million people by the end of 2017. To meet this, Brazil has been forced to request 3.5 million doses of yellow fever vaccine from the International Co–ordinating Group of Vaccine Provision, financed by GAVI. These stockpiles are probably insufficient to meet global demand if there is a major outbreak in cities, where diseases can spread more quickly. Mr Berkley argues that these stockpiles should be the last line of defense, and that better outbreak prevention is vital, in the case of yellow fever via improved mosquito control and immunity against yellow fever through routine immunisation and pre–emptive vaccination campaigns. Preventative approaches can be highly effective, but in order to work, first we must recognize that there is a problem. (New York Times, 15 May 2017)

### The World Bank

➢ According to the World Bank’s Global Economic Prospects report, global economic growth in 2016 was dampened by sluggish performance from the US economy and recession in large commodity–dependent economies, as growth faltered from 2.7% in 2015 to 2.3% in 2016. The Bank forecast a return to 2.7% growth in 2017, supported by the “ripple effect” of US tax cuts and public expenditure promised by the Trump presidency on other developed economies – provided that they are not undermined by aggressive US trade policies pledged during his election campaign. Moreover, rising oil prices are expected to help Brazil, Russia and Nigeria – leading commodity exporters – to move from recession back to growth in 2017. It forecast lower growth rates for the UK economy, as uncertainty over Brexit drags on business and consumer confidence. (The Guardian, 10 January 2017)

➢ The launch of risk–sharing insurance in East Africa is expected to increase the update of agriculture insurance as premiums start to fall. The facility, from the Global Index Insurance Facility (GIIF), and the African Reinsurance Corporation (Africa Re), aims to support regional underwriters to create affordable insurance products, which will enable farmers to be more resilient against external shocks, such as crop failures arising from drought. Across East Africa, uptake of insurance schemes is low, due to existing methods of risk–mitigation and expense – premiums range from 7–15% of the sum insured. The new risk–sharing facility aims to decrease premiums to 4%. According to Mr Makhtar Diop, World Bank Vic President for Africa, insurance expansion is critical for small farmers to build resilience against the impact of climate change. “It is the poor and vulnerable who are the most affected by climate change and natural disasters, and insurance is a critical tool to help protect their livelihoods,” he said. GIIF is managed by the World Bank, with funding from the EU, Japan and the Netherlands. (allafrica.com, 3 March 2017)

➢ The UK’s Department for International Development (DfID) plans to spend half its budget on fragile states and regions; and the World Bank plans to double to US$ 14 billion the money allocated to fragile states over the next 3 years. Up to 33% of the war–scarred Central African Republic’s GDP will comprise World Bank assistance. The accepted wisdom is that aid should be directly at poor, well–governed countries where aid may not be squandered. However, as countries such as India and Vietnam pull more of their people out of poverty, there are fewer such countries and the most acute need is with fragile states with barely–functioning governments. These fragile countries are a regional threat, and if they can be stabilized their neighbors will also benefit. However, it is easy to waste aid money in these places where corruption and mismanagement are rife and infrastructure investments can be destroyed by conflict. Their own systems can be undermined by donors setting up parallel welfare systems, and rich countries often wait until situations become calamitous before rushing in with expensive food aid, when it is often better to give people cash. Another, more intelligent way, is to provide substantial peace–keeping forces to allow the countries to develop and build infrastructures peacefully. Donors could provide risk insurance or subsidies to help private firms enter dangerous markets, and allow governments to set their own spending priorities but channel spending through whatever organisations work in any given area. These are new ideas and could well fail, but may inform future models for fixing failing states. (Economist, 16 March 2017)

➢ The World Bank has cut its forecasts for sub–Sahara Africa’s economic growth in 2017, from 2.9% to 2.6%, which is in line with the International Monetary Fund. The region’s resource–reliant economies, and all commodity exporters, continue to be affected by the slump in prices, which although stabilized remain subdued. South Africa’s credit rating has been downgraded to junk status, and its economy grew by 0.3% in 2016 – the slowest since 2009. The region’s other large economies – Nigeria and Angola – are projected to have faltering economic growth following sharp slowdowns in 2016. However, the World Bank anticipates a rebound, with the region posting growth rates of 3.2% and 3.5% in 2018 and 2019 respectively, although rising interest rates in developed economies and tighter access to finance, sluggish improvements in commodities and a move toward protectionism could threaten this. The Bank also emphasizes the importance of closing Africa’s infrastructure gap – almost US$ 100 billion a year – narrowing it could add up to 2.6% to the region’s GDP each year. (Public Finance Internation, 20 April 2017)

➢ According to the International Labour Organisation (ILO), 40 million jobs per year must be created to keep pace with the growth in the global working–age population. The World Bank notes that the pace of job creation is not keeping pace with the estimated number of entrants into the labour market – between 600 million and 1 billion up to 2030. Economic growth is intrinsic to the Sustainable Development Goals, and is essential for eliminating hunger, improving health care and access to education. Young people make up a large proportion of the world’s unemployed, and the rate is often higher among women. It can be difficult for governments to create work – jobs supported by wage subsidies can disappear when subsidies end, and employers can shed existing workers to recruit subsidised workers. Other tools, such as job guarantees whereby the state promises to hire unemployed workers, are expensive and do not address the structural causes of unemployment. Governments also face the challenge of technologies such as robotics and artificial intelligence wiping out millions of jobs, and there are concerns that governments are unprepared for the impact of the digital economy on the nature of work over the next 10–15 years. (Financial Times, 20 April 2017).

### United Nations

➢ Two UN peacekeepers have been kidnapped in the central Kasai region of the Democratic Republic of the Congo (DRC), with one worker being confirmed as Michael Sharp, a US citizen, and the other as Zaida Catala, a Swedish citizen. They were among a group of UN of experts investigating DRC conflicts, and their kidnappers have not yet been identified. In the preceeding week, a Uruguayan peacekeeper was shot and wounded in the same region, which has fallen victim to a rebellion since September 2016 after government forces killed Kamwina Nsapu, a tribal chief and militia leader. The violence spread to neighboring provinces, leaving at least 400 people dead. Zeid Ra’ad Al–Hussein, the UN High Commissioner for Human Rights, confirmed that three mass graves have been discovered in the area. The UN has almost 19 000 troops deployed in the DRC, its largest and costliest peacekeeping mission. The UN Secretary General, Antonio Guterres, asked the Security Council to send an additional 320 UN police to the DRC, after a deal to end the dispute of the presidential election stuttered. (Al Jazeera, 13 March 2017)

➢ The UN Security Council unanimously renewed its US$ 1.2 billion peace–keeping mission to the Democratic Republic of the Congo (DRC) for another year, albeit at reduced numbers. This renewal comes amid warnings that violence is spreading across the DRC ahead of elections. The resolution authorises the replacement of some troops with better–trained specialist units, and enables the force to intervene anywhere in the DRC if needed, and not just in the volatile east. It also calls for a dialogue between the UN and the DRC government to develop an exit strategy. The UN is pressing DRC’s government to honor a power–sharing deal with the opposition ahead of this year’s election, as violence in the central Kasai region has spilled over into neighboring provinces, leaving at least 400 people dead. There are also reports of violent clashes between Congolese forces and local militia, a large number of deaths, kidnappings and summary executions, all of which could potentially constitute crimes under the International Criminal Court. (Al Jazeera, 31 March 2017)

➢ According to the UN, war and famine have forced more than 2 million children in South Sudan to flee their homes, creating the most troublesome refugee crisis in the world. More than 1 million children have fled outside South Sudan, while another 1 million are internally displaced. And in a country of 12 million people, nearly 75% of children do not attend school. The UN, the UN High Commission for Refugees and UNICEF also report than more than 1000 children have been killed in South Sudan’s civil war, which began 2 years ago following its independence from Sudan. The true figure may be much higher, as there are no accurate figures on deaths from South Sudan. Many of the refugees have fled to Uganda, Kenya, Sudan or Ethiopia – nations who are already struggling to provide enough food and resources for their existing populations. (IRIN, 8 May 2017)

➢ Nikki Haley, the US Ambassador to the UN, confirmed that she will visit Turkey and Jordan, to check on the welfare of Syrian refugees, to see UN humanitarian work and highlight the US aid response. She plans to talk to government leaders about the effectiveness of US programmes to help refugees, and will visit refugee camps and families, US–funded schools, and witness UN efforts to ship humanitarian aid into Syria from Jordan and Turkey. It will be her first official overseas trip, and follows a bid from President Trump to temporarily ban refugees from entering the US and to cut US funding to the UN and its agencies. “What is happening in Syria and its neighbouring countries is a true humanitarian crisis. But those who accuse the US of heartlessness in the face of this crisis are wrong. No country has provided more in protecting, housing, feeding and caring for Syrian refugees that the US. We have provided nearly US$ 6.5 billion in emergency assistance for Syria since the start of the crisis. With American help, Syria’s neighbours have made the difference between life and death for millions of Syrians. The US and UN will continue to do a great deal of heavy lifting for these desperate people,” she wrote in a recent *Wall Street Journal* article. (Business Insider, 17 May 2017)

➢ President Trump confirmed the US’s withdrawal from the Paris climate agreement, arguing that it imposes unfair environmental standards on US businesses and workers, and that it is an attack on US sovereignty, although he reiterated his commitment to the trans–Atlantic alliance and to environmental protection overall. The agreement was intended to bind countries together in a co–operative effort to battle climate change. The US is the world’s second–largest emitter of greenhouse gases, and its withdrawal is a blow toward limiting greenhouse gases and limiting climate change; under the accord it had pledged to cut its greenhouse gases 26–28% below 2005 levels by 2025 and committed up to US$ 3 billion in aid to developing countries by 2020. Some business leaders criticised the decision, claiming it would ultimately harm the economy by ceding future jobs in clean energy and technology to overseas competitors. Mr Trump will stick to the withdrawal process set out in the agreement, which will take nearly 4 years to complete – meaning a final decision on the US withdrawal will be made by American votes in the next presidential election. (New York Times, 1 June 2017)

### UN AIDS and The Global Fund

➢ In partnership with civil society, UN AIDS and the US Agency for International Development, Thailand has developed a stigma reduction programmes that is gradually being rolled out to all public hospitals. It was launched on 2 March 2017, following the annual Zero Discrimination Day of 1 March. The programmes recognizes that the fear of HIV transmission and stigma against people living with HIV hinders access to treatment, care, employment and education, and that tackling this stigma is essential to ending the HIV epidemic. Thailand’s related HIV–stigma reduction programmes for health–care workers is one of the most ambitious in the world, and is being adapted and implemented in other South East Asian countries, including Viet Nam. Other countries such as Laos and Myanmar have expressed interest in similar initiatives, and UN AIDS dedicated the 2017 Zero Discrimination Day to eliminating discrimination in health care settings, to overcome barriers to treatment. (ReliefWeb, 3 March 2017)

➢ According to UNAIDS, 13.9 million people out of the 17 million world–wide on anti–retroviral (ARV) treatment for HIV live in low– and middle–income countries (LMIC). 15 generic manufacturers supply more than 95% of ARVs to LMICs, with 4 suppliers accounting for 83% of the total volume. Moreover, ARVS are largely purchased by 3 buyers – the US President’s Emergency Plan for AIDS Relief (PEPFAR), the Global Fund and South Africa – enabling the rapid expansion of HIV treatment and quality assurance. However, it means that these buyers are largely responsible for treatment continuity, limiting drug resistance and introducing better drug treatments. It is argued that coverage for 30 million people by 2030 [part of the UN AIDS 90–90–90 target, whereby by 2020, 90% of all people living with HIV will know their HIV status; 90% of all people with diagnosed HIV infection will receive sustained antiretroviral therapy; and 90% of all people receiving ART will have viral suppression] must be ensured by these buyers becoming less dependent on a limited number of suppliers, allocating procurement to all suppliers who meet quality standards, and allow excluded or limited suppliers to quickly increase their volume. Eg, PEPFAR procures ARV through annual tenders but the Global Fund and South Africa have 3–year supply contracts, which may be extended and thus exclude other sources – this is hazardous as generic manufacturers of ARVs could switch production to other pharmaceuticals; and all suppliers are vulnerable to supply interruptions and must ensure the continual supply of ARVs to HIV–positive people who have no other treatment options. This over–dependency creates risks because if treatment stops (eg, through breaks in the supply chain), the HIV virus will progress without treatment and there are no treatment alternatives. (Financial Times, 16 March 2017)

➢ UN AIDS has expressed concern at the HIV prevalence in Karachi, and urged Pakistan’s government to devote all available resources to containing it. Mr Mamadou Sakho, UNAIDS Country Director for Pakistan and Afghanistan, reiterated that wider technical and financial support will be available. Mr Sakho noted that HIV is currently concentrated in “at risk” populations, but it may spread and become generalised if it is not tackled within these groups now. Karachi has one of the highest rates of HIV infection in the world, and that the Sindh region has almost 50% of Pakistan’s total HIV population. The Sindha AIDS Control Programme (SACP) will establish more treatment facilities in Karachi and Sindha. It also plans family awareness centers, from basic health untis and rural health centers, up to tertiary levels. These new plans will help SACP to achieve 80% coverage in HIV treatment. (India.com, 27 April 2017)

➢ The Board of the Global Fund met in Kigali, Rwanda, in May and elected Aida Kurtovic as its new Chair. Ms Kurtovic will serve a two–year term as Chair, and she has previously served as the Global Funds’ Vice–Chair, as well as being involved in various other capacities. The Board also elected John Simon, the former US ambassador to the African Union, as the incoming Vice–Chair. Opening the board meeting, the Rwandan President Paul Kagame highlighted the importance of strengthening health systems, aligning financial support with countries’ strategic health plans, and long–term improvement underpinned by constant learning. Statistics from the Rwandan government shows that Global Fund investments in the country have meant that 175 000 people receive ART treatment for HIV, more than 36 000 cases of TB were detected and treated, and more than 18.1 million insecticide–treated nets were provided to protect families from malaria. (Xinhua, 4 May 2017)

➢ Botswana – with an estimated 25% HIV prevalence among its adult population – was the first country in Africa to establish an antiretroviral therapy (ART) program, and is close to achieving the UN AIDS’s 90–90–90 target. According to research published in *Clinical Infectious Diseases*, there is a significantly–higher incidence of cryptococcal meningitis (CM) among HIV–positive people compared to the general population. This suggests that key populations are developing advanced HIV and associated opportunistic infections, as they are not engaging in care. Botswana’s rate of CM among HIV–positive people is similar to pre–ART South Africa, and 30% of Botswana’s people aged 10–64 years had not been tested for HIV in 2013, and that its ART program must reach an additional 100 000 people. The study’s researchers suggested adjusting the program to fit the needs of specific populations: “to avoid leaving vulnerable individuals behind, differentiated care models should be considered to streamline care for populations with well–controlled disease and focus more intensive resources on those with higher need who are now driving the epidemic,” they write. (Healio, 24 May 2017)

### UNICEF

➢ New figures from UNICEF shows that Boko Haram have used 27 children to carry out suicide bombing attacks in the first 3 months of 2017, in Nigeria, Chad, Niger and Cameroon – an increase on the 30 children in 2016. In total, 117 children have been used in suicide attacks since 2014 – 80% of them girls. This move suggests a change in tactics by Boko Haram, as it moves from holding towns and terrority toward a guerilla–style insurgency with hit–and–run attacks and improvised explosives. The group systematically kidnaps children – including the 270 girls taken from Chibok, Nigeria in 2014 – who may be forced or deceived into carrying out suicide attacks. It appears that not all of the children are aware of their actions, and the group’s strategy of using children in suicide attacks makes it more difficult for returning children to be re–integrated into their communities upon release, as they can be suspected of carrying explosives. (NPR, 13 April 2017)

➢ According to Robin Nandy, principal adviser and UNICEF’s head of immunisation, rapid urbanisation presents a massive challenge in vaccinating the world’s poorest children, and increases the risk of rapidly spreading disease outbreaks. More and more unvaccinated children live in urban slums with limited vaccination coverage – especially concerning because disease outbreaks can potentially spread more quickly and infect more people. The UN estimates that 25% of people will live in urban slums by 2030, mainly in Africa and Asia, and the Ebola outbreak shows how quickly outbreaks can spread in cities. The pressure on cities’ immunisation services is increased by growing numbers of refugees settling in urban areas – many of whom have not been vaccinated due to weak or crumbling health infrastructures in their conflict–ridden home areas. Each year, around 19.4 million children – many in war–torn areas – miss out on full vaccinations, while weak health systems, poverty and inequality also lead to 1–in–5 of all children aged under 5 years not being immunised. The poorest children are almost twice as likely to die before 5 years of age compared to richer children, and in those countries accounting for 80% of deaths in children aged under 5 years, over 50% of children are not fully vaccinated. Each year, 1.5 million children die from vaccine–preventable diseases. Mr Nandy also expressed concern that recent outbreaks of measles could indicate a resurgence of the disease, causing unnecessary deaths and illness among children. (Thomson Reuters Foundation, 26 April 2017)

➢ In a report released in May, UNICEF counted 300 000 unaccompanied and separated refugee children globally in 2015 and 2016 – compared to 66 000 in 2010 and 2011. Out of the total, around 100 000 children were trying to cross from Mexico into the USA. Overall, around 200 000 children applied for asylum in 80 countries, including 170 000 lone children – and 92% of children and young people who reached Italy by boat in 2016 traveled alone, compared to 75% in 2015. Some of the children are orphans, others are seeking to join relatives, and the parents of the remainder believed that unaccompanied children would have a greater chance of being allowed to stay. UNICEF called upon the countries where children have sought asylum to provide better services, and not to be placed in adult detention camps. At the G7 summit in May 2017, UNICEF will urge world leaders to protect refugee and migrant children from exploitation, violence and detention, to keep families together, and give children access to education and health care. UNICEF also calls for action on the underlying causes of large–scale migration, and measures to combat xenophobia and discrimination in both transit and destination countries. (Al Jazeera, 18 May 2017)

➢ UNICEF found that up to 150 children aged under 5 years die in Myanmar each day, despite the reform and reconciliation promoted by Aung San Suu Kyi’s one–year government. UNICEF estimates that Myanmar’s child mortality rate is 50 per 1000 live births, compared to 4 per 1000 in the UK, nearly 30% of children under 5 suffer from moderate or severe malnutrition, and that more than 50% of all children live below the poverty line. There are disparities across Myanmar, especially among families in war zones unable to reach health clinics; and UNICEF calls for improved humanitarian access to the estimated 2.2 million children affected by violence. UNICEF acknowledges that Myanmar is undergoing an “unprecedented period of change and opportunity”, but that progress has been disappointingly slow, and there is a worrying escalation of conflict in more remote border areas. In Rakhine province, 120 000 people who have been displaced by violence live in camps, and UNICEF’s humanitarian access to these camps is still highly problematic, although improving slightly. It calls for an end to the laying of landmines, and for landmine clearances to start whenever possible – highlighting that 1–in–3 victims of landmines are children. (The Guardian, 23 May 2017)

➢ UNICEF’s recent study on Thailand’s Comprehensive Sexuality Education (CSE) programmes found that sex education at secondary level does not equip students with the skills needed to manage their sexuality and sex lives, despite near–universal coverage. Much of the programme’s teaching focuses on imparting information, rather than developing critical thinking, and communication and negotiation skills, and important topics such as rights, gender equality and diversity are ignored – risking the development of skewed attitudes toward equality, domestic violence and rights. Effective sex education is vital in reducing Thailand’s high rate of teenage pregnancy, and the high rate of sexually–transmitted diseases among young people. The study also found that 41% of male vocational students thought that it was acceptable for a husband to physically punish his wife for adultery, 50% believed that same–sex relationships are wrong, most students did not know basic facts about the menstrual cycle, and only 54% of female students were confident that they could insist on condom use. The CSE programme was found to focus on biology, abstinence on sex before marriage, and preventing unwanted pregnancy, and 50% of teachers did not receive training on delivering CSE. It concludes by recommending that the program’s delivery includes all designated topics, that it should foster critical thinking, that each school allocates enough time for it, and that every teacher receives full training in it. (The Nation, 1 June 2017)

### World Health Organization (WHO)

➢ The WHO is launching a global initiative to reduce severe and avoidable medication–associated harm by 50% over the next five years. The Global Patient Safety Challenge is the WHO’s third patient safety campaign, following the Clean Care is Safe Care and Safe Surgery Saves Lives initiatives. It aims to address health system weaknesses that lead to medication errors and the resultant harm to patients. According to the WHO, medication errors cause at least one death every day and globally injure 1.3 million people each year. As well as the human cost, this places a huge strain on health budgets and families – at an estimated annual US$ 42 billion, or almost 1% of total health expenditure. Preventing errors, which can be caused by fatigued workers, overcrowding, staff shortages, poor training, lack of co–ordination among agencies and wrong patient information, would save lives and money. “Preventing errors and the harm that results requires putting systems and procedures in place to ensure the right person receives the right medication via the right route at the right time,” said WHO Director–General Margaret Chan. (SunStar, 30 March 2017)

➢ The WHO is set to update its recommendations for treating postpartum hemorrhage, following the results of a study published in *The Lancet*, which suggests that tranexamic acid could cut deaths by one–third. The study was co–ordinated by the London School of Hygiene and Tropical Medicine, in collaboration with 193 hospitals across Africa and Asia. Each year, 100 000 women die from postpartum hemorrhaging, and it is the biggest cause of death during pregnancy and birth. 77% of maternal deaths occur in just 20 countries, and 1360 mothers per 100 000 die in childbirth in Sierra Leone, compared to 3 maternal deaths per 100 000 in Greece, Poland and Finland. Tranexamic acid prevents clots from breaking down, making it easier for the body to stem bleeding. It was developed by husband and wife team Shosuke and Utako Okamoto in 1960s Japan, but they could not persuade pharmaceutical companies to conduct clinical trials for treating postpartum hemorrhage. Tranexamic acid is cheap – US$ 1 per dose – and is given in a single shot, making it easy to administer. Prof Ian Roberts, one of the study’s researchers, admits that making the drug accessible around the world will be a challenge. “When we started the trial, the staff would cry hearing about babies left without their mothers. Making sure the treatment is available everywhere it can save a life is hugely important. We shouldn’t have children growing up without a mother for lack of a drug that costs a dollar,” he says. (BBC, 27 April 2017)

➢ The WHO and Somalia’s Federal Ministry of Health have launched a preventative oral cholera vaccination campaign, targeting 224 000 people aged over 1 year. The campaign follows a major outbreak of cholera in January 2017, with a total of 31 674 reported cases and 618 deaths, mainly in the South West State. Drought has worsened the cholera outbreak, due to shortages of clean water and sanitation. The campaign does not replace other preventative measures, such as clean water and good hygiene, and is supported by the Global Task Force on Cholera Control, GAVI, UNICEF and other health partners. Dr Ghulam Popal, WHO’s representative in Somalia, reiterated WHO’s commitment to support cholera response efforts in Somalia. “We are working with health authorities at all levels and humanitarian partners to limit this outbreak,” he said. (Outbreak News, 7 May 2017)

➢ According to the WHO, improvements in data collection have led to nearly 50% of global estimated deaths being registered with a cause in 2015, compared to 33% in 2005. This means that 27 million of the world’s 56 million deaths were registered with a cause in 2015. Several countries, including China and Turkey, have made significant progress in data collection, and Iran has moved from recording the cause of death in only 5% of cases in 1999 to 50% in 2015. Recording the causes of death enables countries to develop and implement more effective health systems, and address the underlying causes of mortality. The WHO is working with countries to strengthen their health information systems and improve data quality. (Reuters, 17 May 2017)

➢ The WHO announced the appointment of its next director general, Tedros Adhanom Ghebreyeus, who will take over from Dr Margaret Chan. His 5–year term will begin on 1 July, and he will be the first WHO director–general from Africa. The election coincides with a deterioration in the WHO’s reputation over its handling of the 2014 Ebola outbreak. Mr Ghebreyesus is a former health and foreign affairs minister, and according to his application, as Ethiopia’s health minister he oversaw the creation of 3500 health centers and 16 000 health posts, contributing to falls in child mortality, HIV infections, and malaria and tuberculosis deaths. In a pre–election speech, Mr Ghebreyeus spoke of his background of “knowing survival cannot be taken for granted, and refusing to accept that people should die because they are poor.” (Time, 23 May 2017)

